# IL-17 Induces Autophagy Dysfunction to Promote Inflammatory Cell Death and Fibrosis in Keloid Fibroblasts *via* the STAT3 and HIF-1α Dependent Signaling Pathways

**DOI:** 10.3389/fimmu.2022.888719

**Published:** 2022-06-10

**Authors:** Seon-Yeong Lee, A Ram Lee, Jeong Won Choi, Chae Rim Lee, Keun-Hyung Cho, Jung Ho Lee, Mi-La Cho

**Affiliations:** ^1^ Lab of Translational ImmunoMedicine, Catholic Research Institute of Medical Science, College of Medicine, College of Medicine, The Catholic University of Korea, Seoul, South Korea; ^2^ Department of Biomedicine and Health Sciences, College of Medicine, The Catholic University of Korea, Seoul, South Korea; ^3^ Department of Plastic and Reconstructive Surgery, College of Medicine, The Catholic University of Korea, Seoul, South Korea; ^4^ Department of Medical Lifescience, College of Medicine, The Catholic University of Korea, Seoul, South Korea

**Keywords:** keloid, autophagic flux, IL-17, stat3, HIF-1α

## Abstract

Keloid is an abnormal fibrotic disease after cutaneous injury characterized by exaggerated scar tissue formation, which often extends beyond the boundaries of the original wound. Although chronic inflammation is known to be associated with the excessive inflammation in keloid tissue, there are few studies on the role of autophagy in the pathogenesis of keloid. In this study, we evaluated the pattern of autophagy in keloid fibroblasts (KF) and normal fibroblasts (NF). Expression of HIF-1α, STAT3 and autophagic flux markers were evaluated in KF and NF. Defective autophagy caused by IL-17 was evaluated, and the relationship between defective autophagy and necroptosis was also examined. The expression of IL-17, HIF-1α and STAT3 was significantly increased in keloid tissue, and autophagosome-to autophagolysosome conversion was defective in KF. IL-17 treatment significantly elevated the expression of STAT3 and HIF-1α in NF and caused defective autophagy, which was reversed by HIF-1α inhibitor. In addition, the defective autophagy was associated with the increased necroptosis and fibrosis. In keloid tissue, the elevated necroptosis marker was confirmed, and with the HIF-1α inhibitor, the defective autophagy, necroptosis and fibrosis was decreased in KF. In conclusion, autophagy was defective in keloid tissue, which was associated with increased necroptosis and fibrosis. The IL-17-STAT3-HIF-1α axis was involved in defective autophagy in KF, and this suggests that targeting the axis could alleviate chronic inflammation in keloid disease.

## Introduction

Keloid is an abnormal fibrotic disease after cutaneous injury characterized by exaggerated scar tissue formation, which often extends beyond the boundaries of the original wound. As well as being a cosmetic issue, keloid scarring is associated with pain and pruritis, and patient quality of life is reduced (1). Various treatment modalities including surgery, cryotherapy, radiation therapy, and compression therapy have been used for keloid, but it often relapses (2). To correct excessive fibrosis, elucidating the mechanism underlying keloid formation is important.

The mechanism underlying keloid formation is unclear. However, the fibrotic response in keloid results from chronic inflammation, leads to chronic fibroblast activity, and blocks scar maturation (3). Various pro-inflammatory cytokines (TGF-β, interleukin [IL]-4, IL-5, IL-6, and IL-17) play a role in keloid formation (4, 5), by causing excessive extracellular matrix deposition in association with paracrine signals arising from activated immune cells or autocrine signals arising from fibroblasts (6).

There is a relationship between inflammatory diseases and autophagy (7–10). Autophagy is a conserved cellular homeostatic process that regulates the turnover of cellular proteins and removes damaged organelles. In addition, it protects against cytotoxic injury and removes misfolded proteins and damaged organelles (11). Autophagy involves the formation of autophagosomes, which are delivered to lysosomes to become autophagolysosomes. Defective autophagy is linked to various human diseases including fibrotic disorders (6). For example, impaired autophagy decreases the degradation of intracellular collagen, resulting in collagen deposition in the extracellular matrix (12). In addition, inhibition of autophagy promotes the epithelial-mesenchymal transition (EMT) in alveolar epithelial cells and pulmonary fibrosis (13). Hence, chronic inflammation in keloid tissue is associated with altered autophagy in skin fibroblasts. However, there are few studies on the role of autophagy in the pathogenesis of keloid.

We evaluated the pattern of autophagy in keloid fibroblasts (KF) and normal fibroblasts (NF). In addition, we investigated the mechanism of altered autophagy in KF.

## Materials and Methods

### Human Skin Fibroblast Differentiation and Culture

KF were isolated from keloid tissue from six keloid patients, and NF were obtained from residual skin tissue during breast reconstruction surgery. The tissues were digested by collagenase type II (C2-28; Sigma Aldrich, St. Louis, MO, USA). The isolated fibroblasts were cultured with 10% fetal bovine serum (FBS) in Dulbecco’s modified Eagle’s medium (DMEM). For cell signaling analysis, the fibroblasts were cultured with IL-17 (10 ng/mL), hypoxia-inducible factor (HIF)-1α inhibitor (LW6; S8441; Selleck Chemicals, Houston, TX, USA), and hydroxychloroquine (HCQ; 20 μM) for 48 h.

### Histopathological Analysis

Tissue was stained with hematoxylin and eosin (H&E) and Masson’s trichrome to analyze inflammatory cell infiltration and fibrosis. Scarring parameters of circumscription, loss of rete ridges, epidermal thickening, inflammation, abnormal collagen distribution, and vascularity were assigned ordinal scores from 0 (none) to 4 (most severe).

### Immunohistochemistry

Paraffin-embedded sections were incubated at 4°CC with the following primary monoclonal antibodies: anti-human-HIF-1α (MA1-516; Thermo Fisher, Waltham, MA, USA), anti-human- IL-17 (MAB3171-100; R&D Systems, Minneapolis, MN, USA), anti-human-phospho-mixed lineage kinase domain like pseudokinase (*p*-MLKL) (ab187091; Abcam, Cambridge, UK), anti-human-receptor-interacting protein kinase-3 (RIP3) (PA5-19956; Thermo Fisher), and anti-human-MLKL (ab196436; Abcam). The samples were next incubated with the respective secondary biotinylated antibodies, followed by incubation for 30 min with streptavidin–peroxidase complex. The reaction product was developed using 3, 3-diaminobenzidine chromogen (K3468; Dako, CA, Glostrup, Denmark). Immunohistochemistry was performed on sections of skin tissues. Three slides were prepared for each sample and sections were at least 500 µm apart. Immunostained sections were examined under a photomicroscope (Olympus, Tokyo, Japan). The number of positive cells in one high-power field was counted (magnification 400×) using Adobe Photoshop software and the average was calculated for three randomly selected fields per section. Bar graph is optimal density for target protein in the field.

### Western Blotting

Protein concentrations were measured using the Micro BCA™ Protein Assay Kit (23235; Thermo Fisher) and proteins were resolved by 10% or 14% sodium dodecyl sulfate-polyacrylamide gel electrophoresis (SDS-PAGE) and transferred to a nitrocellulose membrane (Amersham Pharmacia, Uppsala, Sweden). The levels of HIF-1α (ab179483; Abcam), signal transducer and activator of transcription 3 (STAT3; #9139; Cell Signaling Technology, Danvers, MA, USA), *p*-STAT3^Tyr705^ (#9145; Cell Signaling Technology), *p*-STAT3^Ser727^ (#9136; Cell Signaling Technology), lysosomal-associated membrane protein 1 (LAMP1; sc-20011; Santa Cruz Biotechnology, Santa Cruz, TX, USA), p62 (ab56416; Abcam), microtubule-associated protein 1 light chain 3 (LC3; ab48394; Abcam), *p*-MLKL (ab184718; Abcam), MLKL (Abcam), and GAPDH (ab181602; Abcam) were detected by Western blotting. Signals were detected using anti-mouse or anti-rabbit HRP-conjugated secondary antibodies. The western blots were quantification using Fiji/imageJ program. Target protein was normalized with GAPDH and, phospho-proteins normalized with total protein and GAPDH.

### Reverse Transcription-Quantitative Real-Time PCR

*Alpha smooth muscle actin (α-SMA)* and *collagen type Iα 1 (COL1A1)* mRNA expression levels were determined by real-time PCR with SYBR Green I (Takara, Shiga, Japan). Reaction mixtures were amplified in a LightCycler (Roche Diagnostics, Mannheim, Germany). Fluorescence curves were analyzed using LightCycler software v. 4.0. Expression levels were calculated and normalized relative to the housekeeping gene (*β-actin*; control). The primer sequences were *α-SMA* (S;5’-TGG GTG ACG AAG CAC AGA GC-3’/AS;5’-CTT CAG GGG CAA CAC GAA GC-3’), *COL1A1* (S;5’- GTC ACC CAC CGA CCA AGA AAC C-3’/AS;5’-AAG TCC AGG CTG TCC AGG GAT G-3’), and *β-actin* (S;5’- GGA CTT CGA GCA AGA GAT GG-3’/AS 5’- TGT GTT GGG GTA CAG GTC TTT G-3’).

### Autophagy Staining and Confocal Laser Scanning Microscopy

LC3, p62, and LAMP1 expression levels were analyzed by confocal microscopy. NF, KF, and IL-17-treated NF (3 × 10^4^ cells) were cultured in Nunc™ Lab-Tek™ II Chamber Slide™ (T460-27; Nunc, Roskilde, Denmark), washed with phosphate-buffered saline, fixed in 4% paraformaldehyde, and blocked with 10% normal goat serum for 30 min. The cells were stained with anti-LC3 (Abcam), anti-LAMP1 (Santa Cruz Biotechnology), and anti-p62 (Abcam) antibodies. Nuclei were stained with DAPI (D3571; Thermo Fisher). Stained cells were analyzed using a confocal microscope (LSM 510 Meta; Carl Zeiss, Oberkochen, Germany). Fluorescence intensity was analyzed using ZEN 2009 software.

### Electron Microscopy

NF and KF were fixed in 4% paraformaldehyde and 2.5% glutaraldehyde in 0.1 M phosphate buffer. The cells were stained with uranyl acetate and lead citrate, and visualized by transmission electron microscopy (TEM; JEM 1010; JEOL, Tokyo, Japan).

### Plasmid and Transfection

*Escherichia coli* containing the pBABE-puro mCherry-EGFP-LC3B vector (#22418; Addgene, Watertown, MA, USA) was incubated in Luria–Bertani high-salt broth (L1704; Duchefa Biochemie, Haarlem, Netherlands) at 37°C. The DNA vector was isolated using a NucleoBond Xtra Maxi EF Kit (740426.50; Macherey-Nagel, Dueren, Germany). NF and KF were seeded in Nunc™ Lab-Tek™ II Chamber Slides™ (3 × 10^4^ cells) with 10% DMEM. pBABE-puro mCherry-EGFP-LC3B DNA vector was transfected into NF and KF using X-treme GENE HP DNA transfection reagent (6366236001; Sigma-Aldrich); after 6 h, the transfected cells were cultured with an HIF-1α inhibitor, HCQ, and IL-17 for 48 h.

### Ethics Statement

This study was approved by the Institutional Review Board of the Catholic University of Korea Bucheon St. Mary’s Hospital (HC18TESI0013).

### Statistical Analysis

Results are means ± standard error of the mean (SEM). Data were analyzed by Student’s *t*-test or the Mann–Whitney U test using Prism 9 software (GraphPad Software Inc., La Jolla, CA, USA). *P* < 0.05 (two-tailed) was considered to indicate significance.

## Results

### STAT3 Induced HIF-1α Expression and Dysfunction of Autophagy Flux in KF

**Figure 1:**
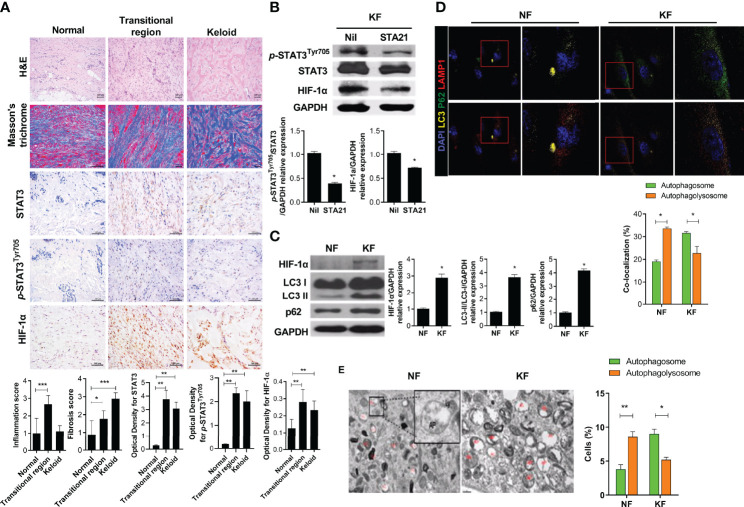
STAT3, HIF-1α expression in keloid tissue and defective autophagy in KF **(A)** Keloid tissues were stained with hematoxylin and eosin (H&E) and Masson’s trichrome (MT), and evaluated by immunohistochemistry. **(B, C)** Protein levels of *p-*STAT3^Tyr705^, STAT3, HIF-1α, LC3I/II, GAPDH and p62 were analyzed using western blot. **(D)** Autophagosomes and autophagolysosomes were analyzed by confocal microscopy. Merging of LC3 (yellow), p62 (green), and DAPI (blue) indicates autophagosomes; Merging of LC3 (yellow), LAMP1 (red), and DAPI (blue) indicates autophagolysosomes. **(E)** Transmission electron micrographs. Bar graph shows data from one of three independent experiments; results are means ± SD of three independent experiments per group (*P < 0.05, **P < 0.01, ***P < 0.001).

STAT3 induces a variety of inflammatory factors, cell survival, cell migration, and inflammatory cytokines (14–16). HIF-1α is induced by STAT3 signaling and promotes cell proliferation and survival in hypoxia (17, 18). The expression of HIF-1α and STAT3 was significantly increased in the transitional region where skin tissue changes into keloid tissue (**Figure 1A**). In addition, inflammatory cell infiltration was significantly increased in the transitional region. Western blot ting showed that the expression of HIF-1α was significantly decreased by a STAT3 inhibitor (STA21), indicating that STAT3 signaling is important for the expression of HIF-1α in KF (**Figure 1B**). Because HIF-1α regulates cell survival, we compared the autophagy process between NF and KF. LC3I conversion to LC3II, an autophagosome marker, was significantly increased in KF compared to NF (**Figure 1C**). However, autophagolysosome formation was decreased in KF according to confocal microscopy and TEM (**Figures 1D, E**). Autophagosomes (LC3^+^p62^+^) were increased in KF, whereas autophagolysosome formation was decreased significantly (LC3^+^LAMP1^+^) according to confocal microscopy. Compared to NF, the number of autophagolysosomes was significantly decreased in KF. Therefore, KF have defective autophagy, as verified by the significantly elevated expression of p62 F (**Figure 1C**).

### IL-17 Increases the Expression of STAT3 and HIF-1α, Leading to Defective Autophagy

**Figure 2:**
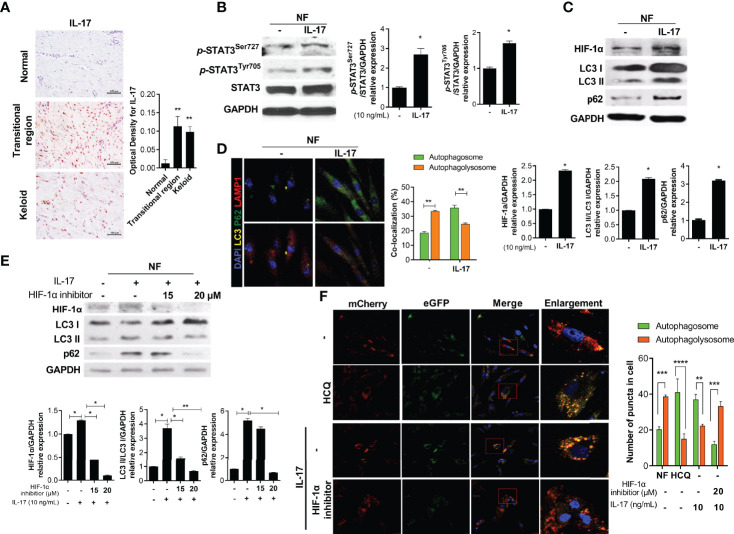
IL-17 promotes activation of STAT3 and HIF-1α, causing defective autophagy. **(A)** Keloid tissues were subjected to IHC for IL-17. **(B, C)** NF was stimulated with IL-17 for 48 h and the protein levels of *p*-STAT3^Tyr705^, *p*-STAT3^Ser727^, STAT3, β-actin, HIF-1α, LC3I/LC3II, p62, and GAPDH were analyzed by Western blotting. **(D)** Autophagosomes and autophagolysosomes in IL-17-treated NF were analyzed by confocal microscopy. Merging of LC3 (yellow), p62 (green), and DAPI (blue) indicates autophagosomes; Merging of LC3 (yellow), LAMP1 (red), and DAPI (blue) indicates autophagolysosomes. **(E)** NF were stimulated with IL-17 (10 ng/mL) and HIF-1α inhibitor (15 or 20 μM) for 48 h. Protein levels of HIF-1α, LC3I/LC3II, p62, and GAPDH were analyzed by Western blotting. **(F)** pBABE-puro mCherry-EGFP-LC3B DNA vector was transfected to NF, which were cultured with IL-17 and an HIF-1α inhibitor (20 μM) or HCQ (20 μM) for 48 h. Autophagosomes (bright yellow) and autophagolysosomes (bright red) were analyzed by confocal microscopy. Bar graph shows data from one of three independent experiments; results are means ± SD of three independent experiments per group (*P < 0.05, **P < 0.01, ***P < 0.001, ****P < 0.0001).

IL-17 expression is increased in KF, inducing the expression of STAT3, stromal cell-derived factor 1 (SDF-1), transforming growth factor-beta (TGF-β), and α-SMA (5, 19). IL-17-expressing cells significantly infiltrated the transitional region of keloid tissue (**Figure 2A**). We cultured NF (2 × 10^4^) with IL-17 (10 ng/mL) for 48 h with serum. IL-17 increased the protein levels of *p*-STAT3^Tyr705^, *p*-STAT3^Ser727^, and HIF-1α (**Figures 2B, C**). Notably, IL-17 increased the expression of LC3II and p62 in NF, indicating autophagosome (LC3^+^p62^+^) accumulation (**Figures 2C, D**). Subsequently, to evaluate whether the defect in delivery of autophagosomes to autophagolysosomes was dependent on HIF-1α, NF (2 × 10^4^) were cultured with 10 ng/mL IL-17 and 15 or 20 μM HIF-1α inhibitor for 48 h. The LC3II and p62 levels were decreased by the HIF-1α inhibitor (**Figure 2E** and **Supplement Figure 1**). Therefore, IL-17 induced defective autophagy in NF, in a manner mediated by HIF-1α. NF (3 × 10^4^) were transfected with pBABE-puro mCherry-EGFP-LC3B DNA vector and cultured with IL-17 (10 ng/mL), with or without 20 μM HIF-1α inhibitor or HCQ for 48 h. HCQ inhibits the delivery of autophagosomes to autophagolysosomes and is known to the induce defective autophagy. The number of autophagosomes was increased significantly in the HCQ and IL-17 culture, while the level of autophagolysosomes was decreased (**Figure 2F** and **Supplement Figure 1**). Therefore, IL-17 caused defective autophagy. The HIF-1α inhibitor reversed IL-17-induced defective autophagy, consistent with the Western blotting results.

### Defective Autophagy Promotes Necroptosis and Fibrosis

**Figure 3:**
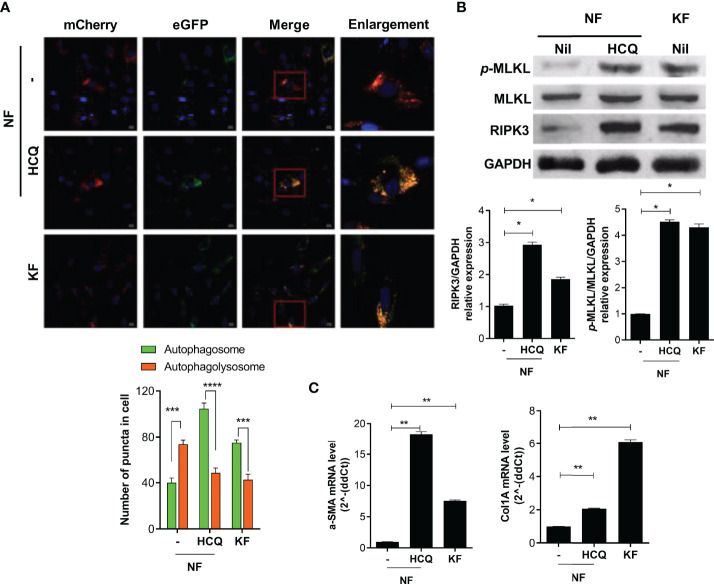
Defective autophagy is associated with necroptosis and fibrosis. NF and KF were transfected with pBABE-puro mCherry-EGFP-LC3B, and NF were cultured with HCQ (20 μM) for 48 h. **(A)** Autophagosomes (bright yellow) and autophagolysosomes (bright red) were analyzed by confocal microscopy. **(B)** Protein levels of *p*-MLKL, MLKL, RIPK3 and GAPDH in HCQ-treated NF and KF were analyzed by Western blotting. **(C)** mRNA levels of *α-SMA* and *Col1A* were analyzed by real-time PCR. Bar graph shows data from one of three independent experiments; results are means ± SD of three independent experiments per group (*P < 0.05, **P < 0.01, ***P < 0.001, ****P < 0.0001).

To evaluate the effect of defective autophagy on necroptosis and fibrosis, we cultured NF (2 × 10^4^) with HCQ (10 μM) for 48 h and evaluated autophagy by mCherry assay. The pattern of autophagy in the presence of HCQ was similar to that of KF (**Figure 3A**). In addition, the expression of necroptosis (RIP3 and *p*-MLKL) and fibrosis markers was significantly elevated by HCQ (**Figures 3B, C**). Therefore, defective autophagy in KF is associated with the increased inflammatory cell death induced by necroptosis and fibrosis.

### IL-17 Induces Inflammatory Cell Death *via* HIF-1α

**Figure 4:**
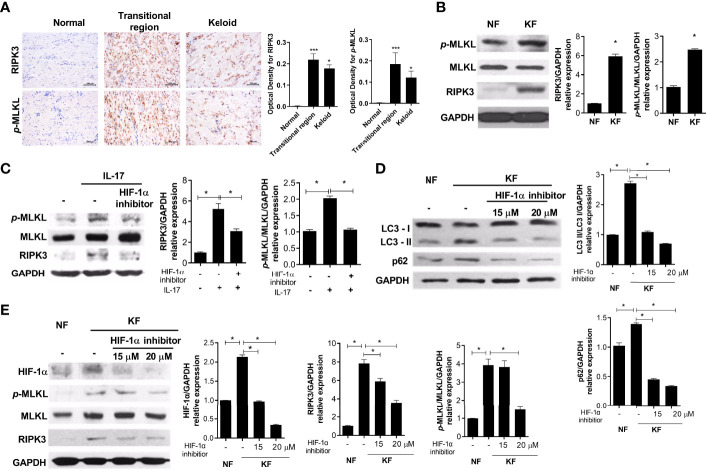
IL-17 induced necroptosis and fibrosis *via* HIF-1α. **(A)** RIPK3 and *p*-MLKL were analyzed by IHC in keloid tissues. **(B)** Protein levels of *p*-MLKL, MLKL, RIPK3, and GAPDH in NF and KF. **(C)** NF was cultured with IL-17 (10 ng/mL) and HIF-1α inhibitor (20 μM) for 48h. Indicated proteins were detected using western blot. **(D, E)** Protein levels of LC3I, LC3II, p62, HIF-1α, *p*-MLKL, MLKL, RIPK3 and GAPDH were analyzed in lysates of NF and HIF-1α-inhibitor-treated KF. Bar graph shows data from one of three independent experiments; results are means ± SD of three independent experiments per group (*P < 0.05, ***P < 0.001).

RIP3- and *p*-MLKL-positive cells significantly infiltrated the transitional region of keloid tissue (**Figure 4A**). In addition, the basal levels of necroptosis proteins were increased in KF compared to NF (**Figure 4B**). NF (2 × 10^4^) were cultured with IL-17 (10 ng/mL), with or without 20 μM HIF-1α inhibitor, for 48 h. The IL-17-mediated increased RIP3 and *p*-MLKL expression was decreased by the HIF-1α inhibitor (**Figure 4C**). Also, the LC3II and p62 levels were decreased in KF treated with the HIF-1α inhibitor, and the expression of RIP3 and *p*-MLKL was decreased by HIF-1α (**Figures 4D, E**). Therefore, targeting HIF-1a in KF could decrease necroptosis and alleviate defective autophagy.

### IL-17-Induced Fibrosis Was Suppressed by the Inhibition of HIF-1α Signaling

**Figure 5:**
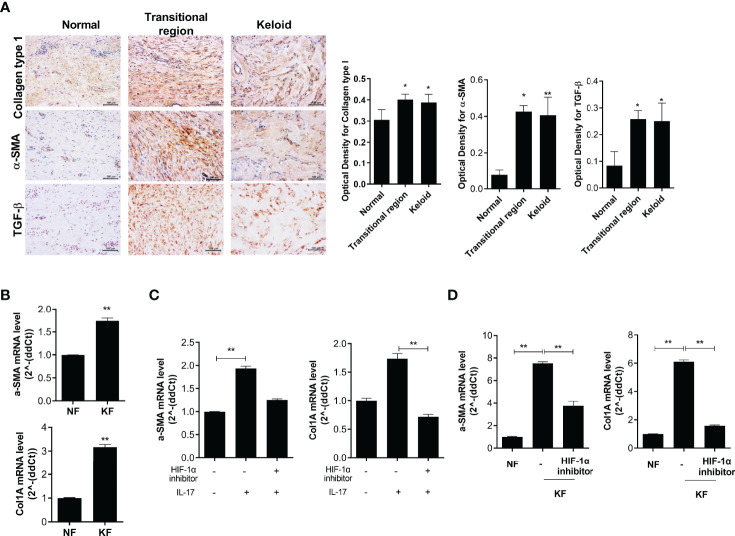
Fibrosis was suppressed by HIF-1α inhibition in KF. **(A)** COL1-, α-SMA-, and TGF-β-positive cells in keloid tissue were analyzed by IHC. **(B)** mRNA levels of *α-SMA* and *Col1A* in NF and KF. **(C, D)** mRNA levels of *α-SMA* and *COL1A* were analyzed in IL-17- and HIF-1α inhibitor-treated NF or NF and HIF-1α inhibitor-treated KF. Bar graph shows data from one of three independent experiments; results are means ± SD of three independent experiments per group (*P < 0.05, **P < 0.01).

**Figure 6:**
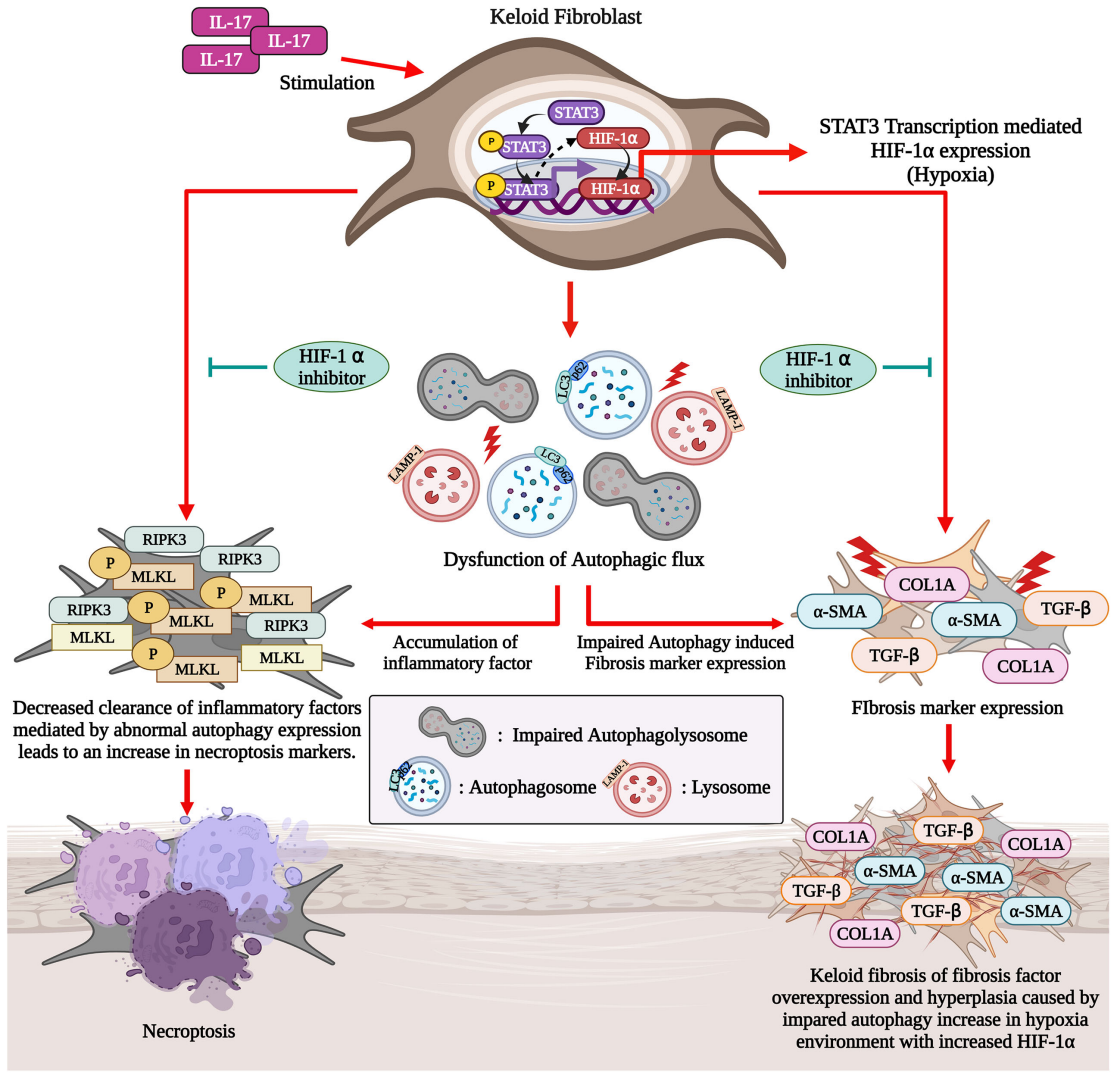
IL-17-activated STAT3 and HIF-1α signaling induces defective autophagy and necroptosis in KF (Graphic Created with BioRender.Com).

The levels of fibrosis markers, such as Col1, α-SMA and TGF-β, were increased in the transitional region of keloid tissue and the expression levels of fibrotic markers (*α-SMA* and *Col1A*) were significantly elevated in KF (**Figures 5A, B**). NF and KF (2 × 10^4^) were cultured with 20 μM HIF-1α inhibitor or IL-17 and *α-SMA* and *Col1A* expression was analyzed. The expression of these genes was increased by IL-17 and decreased by the HIF-1α inhibitor in NF (**Figure 5C**). Also, the expression of *α-SMA* and *Col1A* was significantly suppressed by HIF-1α inhibition in KF (**Figure 5D**). Therefore, fibrosis and inflammatory cell death were inhibited by regulation of autophagy flux under HIF-1α inhibition (**Figure 6**).

## Discussion

In the present study, we have shown that there is a defect in the autophagy of KF, associated with increased necroptosis and fibrosis. The IL-17-STAT3-HIF-1α axis was involved in defective autophagy and increased necroptosis in dermal fibroblasts. Moreover, the inhibition of HIF-1α signaling reversed the changes in autophagy and necroptosis caused by IL-17. These findings suggest that blockade of the HIF-1α pathway has therapeutic potential for keloid disease. We report for the first time that the IL-17-STAT3-HIF-1α axis is involved in defective autophagy and increased necroptosis in skin fibroblasts.

Autophagy refers to lysosomal degradation of cellular components, and is important in cellular homeostasis and survival. For example, during starvation, autophagy provides materials for macromolecular synthesis. When a cell is damaged, autophagy acts as an intracellular recycling process to limit oxidative stress (20). Autophagy is a multi-step process. Under normal conditions, LC3 is localized to the cytosol and translocates to the autophagosomal membrane when autophagy is initiated. Conversion of LC3-I to LC3-II correlates with autophagosome formation, so the LC3-II/LC3-I ratio is an indicator of autophagic activity (21).

Okuno et al. reported increased pan-cathepsin expression and LC3 puncta formation in KF (22). Similarly, Jeon et al. demonstrated increased Beclin 1 and LC3 expression in KF compared to NF. In addition, autophagosomes were increased significantly in KF, suggesting increased autophagic activity (23). In this study, LC3 and autophagosome expression were increased in KF according to TEM and immunohistochemistry. However, autophagolysosome formation, the final step of autophagy, was decreased compared to NF. Therefore, the final step of autophagy is defective in keloid tissue. Furthermore, p62 expression was significantly increased in KF compared to NF. p62 is an autophagic receptor that recruits substrates into the autophagosome. During normal autophagy, p62 and substrates are degraded in autophagolysosomes, and the p62 level is inversely correlated with autophagic activity. Therefore, the increased autophagosome number in keloid tissue represents autophagosome accumulation secondary to defective autophagy (lysosomal degradation of the autophagosome), as opposed to increased autophagic activity.

There is a correlation between defective autophagy and excessive inflammation and fibrosis. Defective autophagy hampers inflammasome clearance, possibly leading to excessive inflammation. In cardiomyocytes, the accumulation of misfolded proteins due to defective autophagy is associated with cardiac fibrosis and ventricular dysfunction (24). In mouse mesangial cells, inhibition of autolysosomal protein degradation results in increased intracellular collagen, and vice versa. In addition, after myocardial infarction, NOD-like receptor pyrin domain-containing protein 3 (NLRP-3) inflammasome forms in cardiac fibroblasts, exacerbating damage to cardiac tissue and causing fibrosis *via* the TGF-beta/SMAD pathway, which can lead to reactive oxygen species and inflammasome accumulation (25). Lee et al. reported that NLRP3 expression was increased in keloid tissue (7).

Necroptosis was also increased in KF. Necroptosis is similar to necrosis but is regulated by RIP1, RIP3 and their substrate, mixed-lineage kinase domain-like (MLKL). In this study, RIP1, RIP3, and *p*-MLKL expression in KF was significantly elevated compared to NF. Necroptosis is a major source of inflammation in acute kidney, heart, and lung injury, and necroptosis-associated proteins regulate inflammation and fibrosis (26) For example, necroinflammation driven by RIPK3-MLKL-dependent necroptosis promotes the progression of acute kidney injury to chronic kidney disease (27). The RIP1/RIP3/MLKL/caspase-8 axis is linked to the posttranslational regulation of NLRP3 inflammasome assembly (28). Therefore, increased necroptosis may be a key mechanism in keloid disease.

The balance among cell death mechanisms was disrupted in KF, contributing to chronic inflammation in keloid tissue. In other words, autophagic cell death was inhibited in keloid tissue, whereas necroptosis was increased. The crosstalk between autophagy and necroptosis is unclear. However, in renal cell carcinoma cells, induction of autophagy suppressed RIP kinase-dependent necroptosis by promoting degradation of RIP1 and RIP3 (20). In this study, necroptosis of NF was significantly elevated upon inhibition of autophagosome-to-autophagolysosome conversion by HCQ. These findings imply that correction of defective autophagy in keloid tissue would decrease inflammation. Indeed, HIF-1α inhibition ameliorated defective autophagy and necroptosis in KF.

The mechanism underlying the defective autophagy seen in KF is unknown. The IL-17 level is increased in keloid tissue (5), and IL-17 is reportedly involved in the chronic inflammation and increased fibrosis seen in keloid tissue (29, 30). We hypothesized that IL-17 signaling modulates autophagy in skin fibroblasts. Indeed, IL-17 altered autophagy and necroptosis of NF such that they were similar to those of KF. In more detail, IL-17 inhibited autophagolysosome formation and increased RIP-1 and -3 expression.

The increased fibrosis caused by IL-17 in KF is dependent on the STAT3 pathway (5). Because HIF-1α regulates autophagy and is modulated by STAT3 (17), we evaluated the IL-17 signaling pathway. IL-17 induced HIF-1α in dermal fibroblast in a manner dependent on the STAT3 pathway. In addition, HIF-1α induced defective autophagy and increased necroptosis in dermal fibroblasts, effects that were reversed by an HIF-1α inhibitor. These findings suggest that IL-17 causes defective autophagy in a manner mediated by the STAT3-HIF-1α axis. This hypothesis was supported by the finding that HIF-1α inhibition decreased necroptosis and defective autophagy in KF. HIF-1α expression is correlated with the expression of proinflammatory cytokines (IL-1β, IL-6, and TNF-α) in KF (31). In addition, collagen production and the epithelial-to-mesenchymal transition in keloid are regulated by HIF-1α, and blockade of HIF-1α suppresses the fibrotic reaction in keloid tissue (32, 33). To our knowledge, this is the first report of a role for HIF-1α in defective autophagy in KF; our findings suggest that HIF-1α blockade may have therapeutic potential for keloid.

## Conclusion

Autophagy was defective in keloid tissue, which was associated with the increased necroptosis and fibrosis. The IL-17-STAT3-HIF-1α axis was involved in defective autophagy in KF, and HIF-1α inhibition decreased necroptosis and defective autophagy in KF. This suggests that targeting IL-17-STAT3-HIF-1α could alleviate chronic inflammation and fibrosis in keloid disease.

## Data Availability Statement

The raw data supporting the conclusions of this article will be made available by the authors, without undue reservation.

## Ethics Statement

This study was approved by the Institutional Review Board of the Catholic University of Korea Bucheon St. Mary’s Hospital (HC18TESI0013). The patients/participants provided their written informed consent to participate in this study.

## Author Contributions

ARL, SYL, JHL, and MLC designed the experiments, analyzed the data and wrote the manuscript. The data performed all in vitro with ARL and CRL. JWC conducted all immunohistochemistry experiments. KHC drew the summary figure. All authors critically reviewed and approved the final form of the manuscript.

## Funding

This work was supported by the National Research Foundation of Korea (NRF) grant funded by the Korea government (MSIT) (No. 2020R1F1A1075541) and Basic Science Research Program through the National Research Foundation of Korea (NRF) funded by the Ministry of Education (grant number 2021R1I1A1A01056024).

## Conflict of Interest

The authors declare that the research was conducted in the absence of any commercial or financial relationships that could be construed as a potential conflict of interest.

## Publisher’s Note

All claims expressed in this article are solely those of the authors and do not necessarily represent those of their affiliated organizations, or those of the publisher, the editors and the reviewers. Any product that may be evaluated in this article, or claim that may be made by its manufacturer, is not guaranteed or endorsed by the publisher.
