# Supplementary material for: IL-17 Induces Autophagy Dysfunction to Promote Inflammatory Cell Death and Fibrosis in Keloid Fibroblasts via the STAT3 and HIF-1α Dependent Signaling Pathways
Source: Front Immunol. 2022 Jun 10;13:888719. doi: 10.3389/fimmu.2022.888719 (PMC9226909; doi:10.3389/fimmu.2022.888719)
Supplement: Supplementary file 1 [file DataSheet_1.docx]

# Supplementary Figure and legends


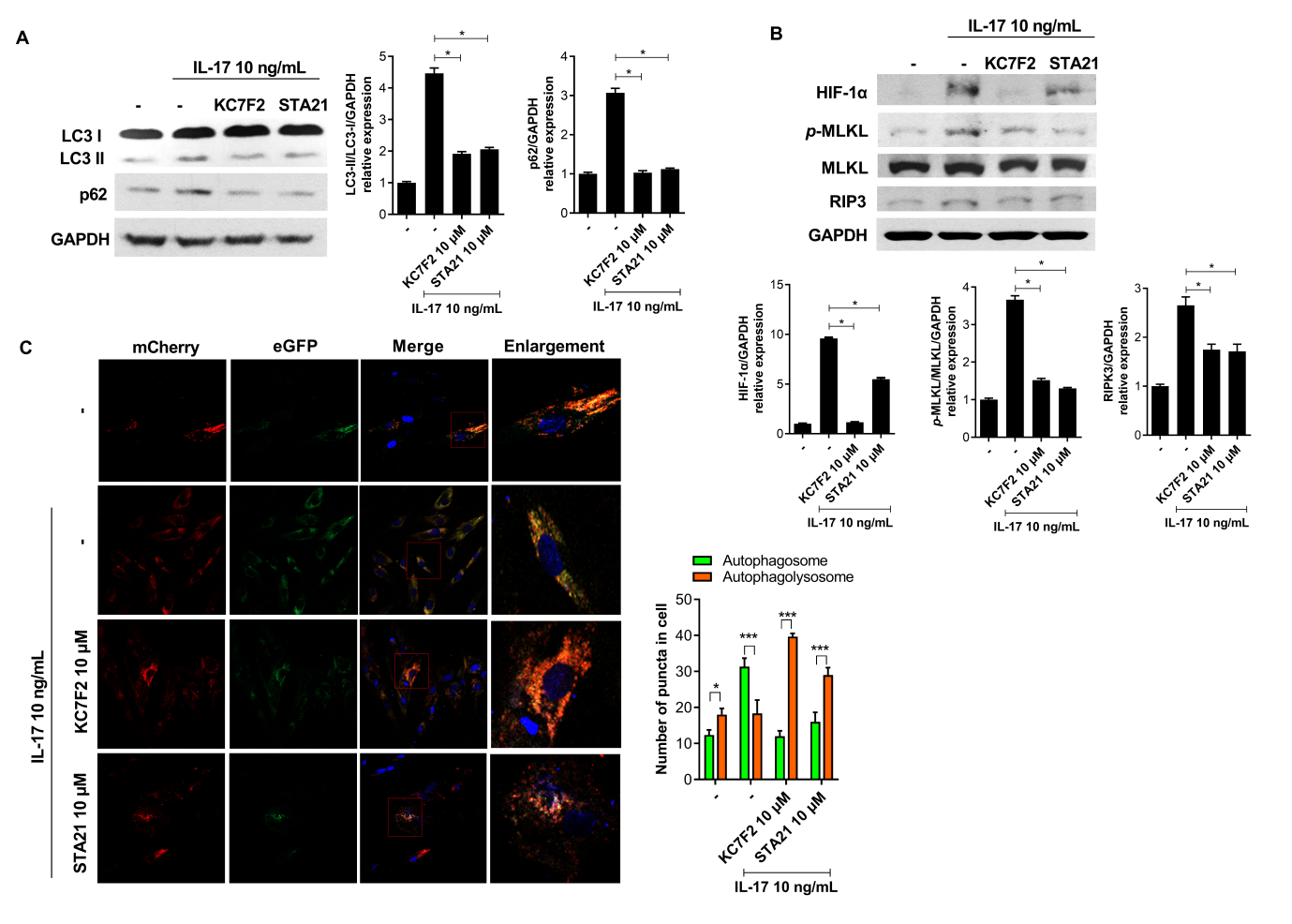


**Supplementary Figure 1. Inhibition of HIF-1α or STAT3 reversed IL-17-induced defective autophagy.** (A and B) NF were cultured with IL-17 (10 ng/mL) and HIF-1α inhibitor, KC7FC2 (10 μM) or STA21 (10 μM) for 48 h. Protein levels of LC3I/LC3II, p62, HIF-1α, *p*-MLKL, MLKL, RIP3 and GAPDH were analyzed by Western blotting. (C) pBABE-puro mCherry-EGFP-LC3B DNA vector was transfected to NF, which were cultured with IL-17 and an HIF-1α inhibitor or STAT3 inhibitor for 48 h. Autophagosomes (bright yellow) and autophagolysosomes (bright red) were analyzed by confocal microscopy. Bar graph shows data from one of three independent experiments; results are means ± SD of three independent experiments per group (**P* < 0.05, ****P* < 0.001).
